# Coordinated Behaviour in Pigeon Flocks

**DOI:** 10.1371/journal.pone.0140558

**Published:** 2015-10-20

**Authors:** Makoto Yomosa, Tsuyoshi Mizuguchi, Gábor Vásárhelyi, Máté Nagy

**Affiliations:** 1 Department of Mathematical Sciences, Osaka Prefecture University, Osaka, Japan; 2 Department of Biological Physics, Eötvös University, Budapest, Hungary; 3 MTA-ELTE Statistical and Biological Physics Research Group of the Hungarian Academy of Sciences, Budapest, Hungary; 4 Department of Zoology, University of Oxford, Oxford, United Kingdom; 5 Department of Collective Behaviour, Max Planck Institute for Ornithology, Konstanz, Germany; Centre for Ecological and Evolutionary Studies, NETHERLANDS

## Abstract

We analysed pigeon flock flights using GPS trajectory data to reveal the most important kinematic aspects of flocking behaviour. We quantitatively investigated the internal motion of the flock based on pairwise statistics and found the following general relationships in all datasets: i) the temporal order of decisions characterised by the delay between directional changes is strictly related to the spatial order characterised by the longitudinal relative position within the flock; ii) during circling motion, pigeons use a mixture of two idealised and fundamentally different turning strategies, namely, parallel-path and equal-radius type turning. While pigeons tend to maintain their relative position within the flock on average, as in the parallel-path approximation, those who turn later also get behind as in the equal-radius case. Equal-radius type turning also tends to be expressed more during smaller radius turns.

## Introduction

Groups of animals often exhibit collective motion which has fascinated observers for a long time [1]. A number of studies have been conducted on flocking behaviour in many research fields, such as biology, ethology, and statistics. Transitions between different collective states have been also studied using statistical physical and mathematical models [1, 2].

Trajectory data have been recorded for various species, including slime molds [3], fish [4], insects [5] and even for birds. Obtaining detailed experimental data from collectively flying birds is challenging. However, recently there have been several related studies using Global Positioning System (GPS) or stereo camera systems [6–11].

One aspect of collective motion is group decision making. For example, homing pigeons in a flock coordinate their behaviour and choose a common route to their home loft. Several numerical [12] and experimental [13, 14] studies have been performed on the subject of collective navigation. Nagy et al. calculated the delay between directional changes of individual pigeons using correlation functions and analysed the leader-follower relationships, i.e. the temporal order of motional decisions in flocks [8].

Kinematic analyses of flight data for several kinds of bird flocks shed light on their spatio-temporal structure during flight. For example, in starling flocks there is an avoidance region behind individuals [6] which is consistent with the disadvantageous aerodynamic effect of downwash air flow. Moreover, a preferred region with upwash flow was observed in skeins of wild geese [7], hooded gull flocks [10], and ibis flying in formation [15].

These features are related to the relative positions of individuals within the flock, which are inevitably constrained by geometric and physical properties of flight orbits during circling motion, such as the range of speed, angular velocity, curvature, or reaction time. Besides these external constraints, birds might also have individual, heterogeneous behavioural preferences for their orbits. Taking all these effects into account, two possible key strategies emerge for control.

In the literature, “equal radii paths” (from here denoted as E type turning) is known as a model of ideal orbits [16, 17]. During E type turning, individuals flying with a common constant speed turn with a common curvature radius simultaneously, as if they were points on a rigid body with translational motion only. In this ideal case, individuals travel exactly the same path length, but their relative positions within the flock vary (the flock rotates around its centre of mass during turning.)

There is another type of ideal orbit, “parallel paths” (from here denoted as P type turning) [18–20]. In this case, relative positions within the flock are maintained and the orbits do not intersect, i.e. the polarized flock behaves as points on a rigid body that maintains its heading towards the direction of motion. To maintain this formation, lengths of individual paths have to be different, i.e. birds have to travel with different velocities.

In order to clarify the actual turning behaviour in flocks, various aspects of birds’ orbits shall be analysed using pairwise comparisons. In this paper, we re-analysed tracking data from pigeon flocks to reveal the spatio-temporal structure of the flock formation. We focused on local, instantaneous relations between individuals rather than e.g. the global, long-term hierarchical order as in [8].

Consistently with [8], we found that there is a tendency to maintain longitudinal relative position when the flock turns. Moreover, we could quantitatively clarify the relation between this longitudinal relative position and the delay of directional changes the same way as for hooded gulls [10].

We also analysed the internal rotational motion of individuals relative to the flock motion to find out whether pigeons use E or P type turning strategies.

## Material

In this paper we re-analysed two datasets from previous studies [8, 21], which contained GPS trajectories of pigeon flocks flying around their nest (see S1 and S2 Videos). Here the first dataset [8] will be referred to as D1. The second study [21] was composed of three datasets, referred to as D2A, D2B and D2C. Note that all of the data used here was from *free* flights, i.e., spontaneous flights near the home loft. Data from *homing* flights was not analysed here, except in one section of the Discussion. There are many differences between datasets D1 and D2, such as sampling frequency, location, training history, regularity of orbits, etc. The specifications of the datasets are summarised in Table 1. Also note that an interesting behavioural difference between datasets D1 and D2 is that while the D1 flocks spend approximately the same amount of time in clockwise and counter-clockwise orbits, D2 flights are generally biased towards CW directional circles. D1 flights are also more regular in terms of the steadiness of the angular velocity (see the electronic supplementary material, S1 Fig).

**Table 1:** Differences between D1 and D2 datasets.

dataset	D1	D2A	D2B	D2C
number of releases	11	5	5	5
total flight log duration (s)	1.43 × 10^4^	0.56 × 10^4^	0.33 × 10^4^	0.50 × 10^4^
age of subjects (year)	2.1 ± 1.5 (1 − 5)	3.3 ± 1.8 (1.5 − 5.5)	3.3 ± 1.8 (1.5 − 5.5)	1.7 ± 0.8 (0.5 − 2.5)
average number of airborne pigeons per release	4.6 ± 5.1	7.5 ± 7.6	5.8 ± 6.0	7.1 ± 7.3
number of subjects in dataset	13	10	10	10
sampling frequency (Hz)	5	10		
location	urban area in Budapest	country area near Oxford		
date of measurement	2008 summer	2010 winter		
living conditions	racing pigeons	free-ranging domestic pigeons		

Each dataset consists of flock flights of 10 pigeons in multiple releases. A maximum of two releases were conducted per day. Age of the flock members is expressed as the average and standard deviation. Age range is shown in brackets. The number of airborne pigeons per release (mean ± S.D.) shows how many individuals actually flew together in a flock. There was no overlap in membership between the flocks in different datasets.

**Fig 1:**
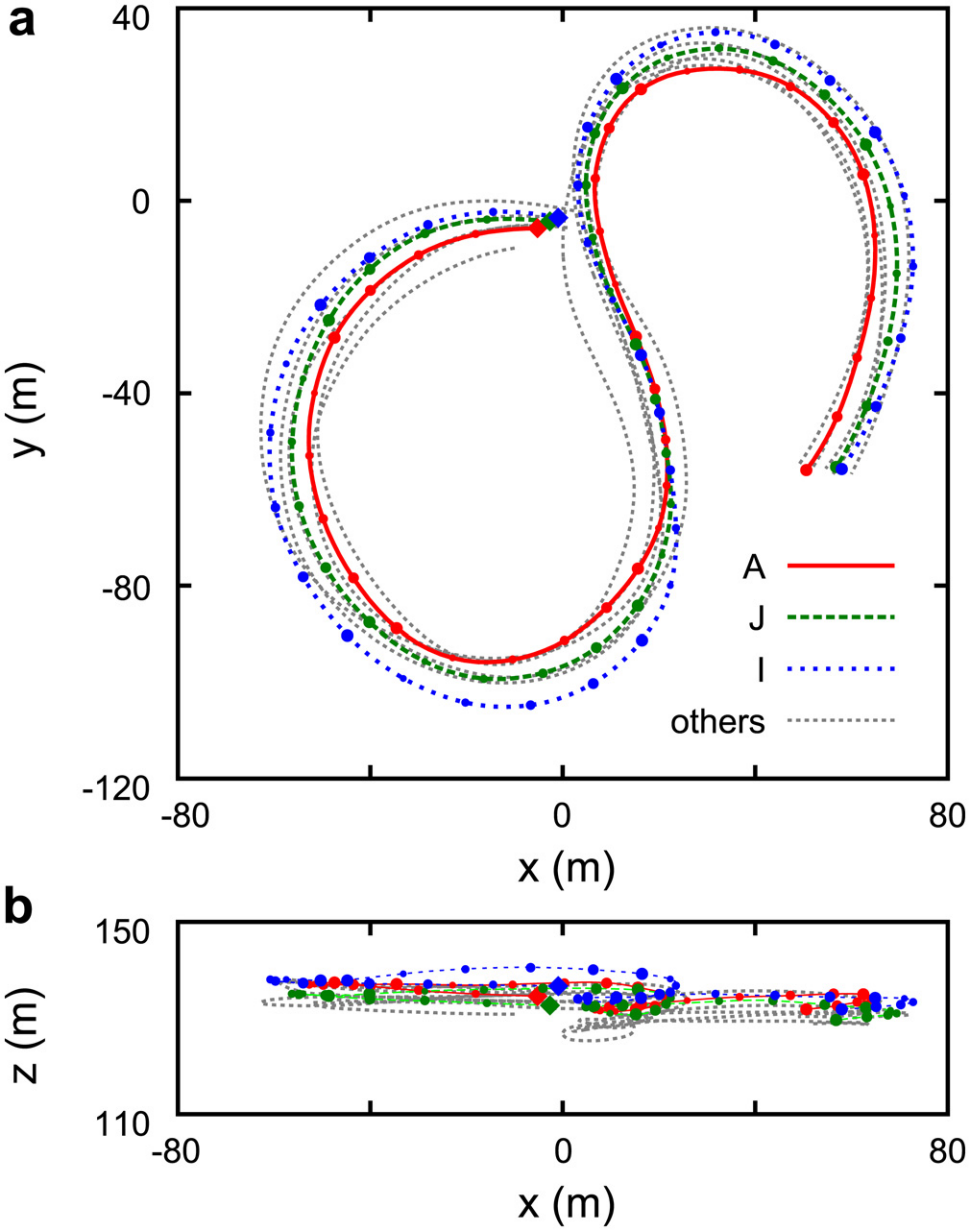
Typical trajectories of eight individuals (dataset D1). Top view (a) and side view (b). Individuals A, I and J are emphasised by thick coloured lines as a typical example of coordinated behaviour. Diamonds denote the starting points (close to (0, 0)). The time interval between points is 1 s. The symbol size increases with time and is reset to small every 5 s.

Fig 1 shows the typical trajectories of eight members of dataset D1. Individuals A, I, and J are emphasised by thick lines as they typify the characteristic motion we are focusing on in this paper. Flock members move collectively, mostly orbiting around their home loft, and they sometimes change their circling direction. In Fig 1, they start from the positions denoted by diamonds. First, they make a turn counter-clockwise then they change direction to clockwise. Note that the height variation of each orbit is considerably narrower than the horizontal range (see the electronic supplementary material, S2 Fig).

To characterize the structure of the flights, we introduce several quantities. Let the three-dimensional position of the *i*-th individual at time *t*_*n*_ be ${\vec{r}}_{i}\left(t_{n}\right)=\left(r_{i}^{x}\left(t_{n}\right),r_{i}^{y}\left(t_{n}\right),r_{i}^{z}\left(t_{n}\right)\right)$ expressed in geocentric coordinates where $r_{i}^{x}$ and $r_{i}^{y}$ are horizontal flat-earth coordinates and $r_{i}^{z}$ represents the height above mean sea level. We calculated the velocity of the *i*-th individual, ${\vec{v}}_{i}\left(t_{n}\right)=\left(v_{i}^{x}\left(t_{n}\right),v_{i}^{y}\left(t_{n}\right),v_{i}^{z}\left(t_{n}\right)\right)$ by taking the discrete derivative of the successive snapshots of ${\vec{r}}_{i}\left(t_{n}\right)$. Here, $v_{i}=\left|{\vec{v}}_{i}\right|$ is the velocity amplitude and $\omega_{i}={\dot{\theta }}_{i}$ is the horizontal angular velocity of the *i*-th individual ($\theta_{i}\equiv \tan^{-1}(v_{i}^{y}/v_{i}^{x})$).

**Fig 2:**
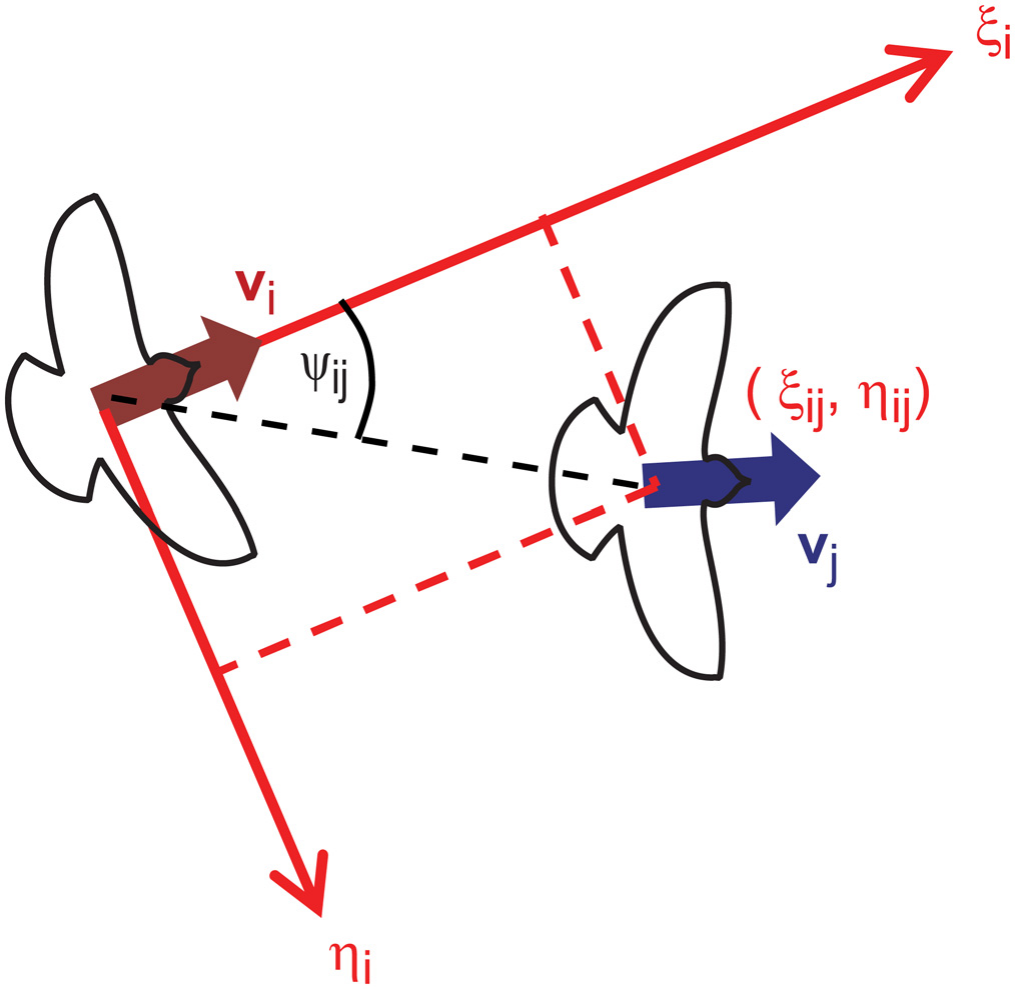
Illustration of the individual coordinate system. The longitudinal (*ξ*_*i*_) and lateral (*η*_*i*_) axes are determined by the momentary motion of the *i*-th individual. (*ξ*_*ij*_, *η*_*ij*_) denote the coordinates, *ψ*_*ij*_ the polar angle of the *j*-th individual in the *ξ*_*i*_ − *η*_*i*_ plane. This figure is a modified version of Fig 3 of [10].

We introduce an *individual coordinate system* that moves with each bird (Fig 2). The longitudinal axis (*ξ*_*i*_) is parallel to the direction of motion of the *i*-th individual (pointing forward). In general, this axis may have a vertical component relative to the ground, but in the datasets analysed in this article (S2 Fig), the motion is mainly restricted to the horizontal plane. The lateral axis (*η*_*i*_) is horizontal and orthogonal to the *ξ*_*i*_-axis (pointing right, horizontally). The third axis (*ζ*_*i*_) is orthogonal to both *ξ*_*i*_ and *η*_*i*_, pointing almost downwards. We define *individual coordinates* *ξ*_*ij*_, *η*_*ij*_ and *ζ*_*ij*_ representing the relative position of individual *j* in the coordinate system of individual *i*, i.e. ${\vec{r}}_{j}-{\vec{r}}_{i}$, expressed in *ξ*_*i*_, *η*_*i*_ and *ζ*_*i*_. Thus the relative position of individual *j* in the coordinate system of individual *i* can be characterised by the relative polar angle *ψ*_*ij*_ ≡ tan^−1^(*η*_*ij*_/*ξ*_*ij*_).

**Fig 3:**
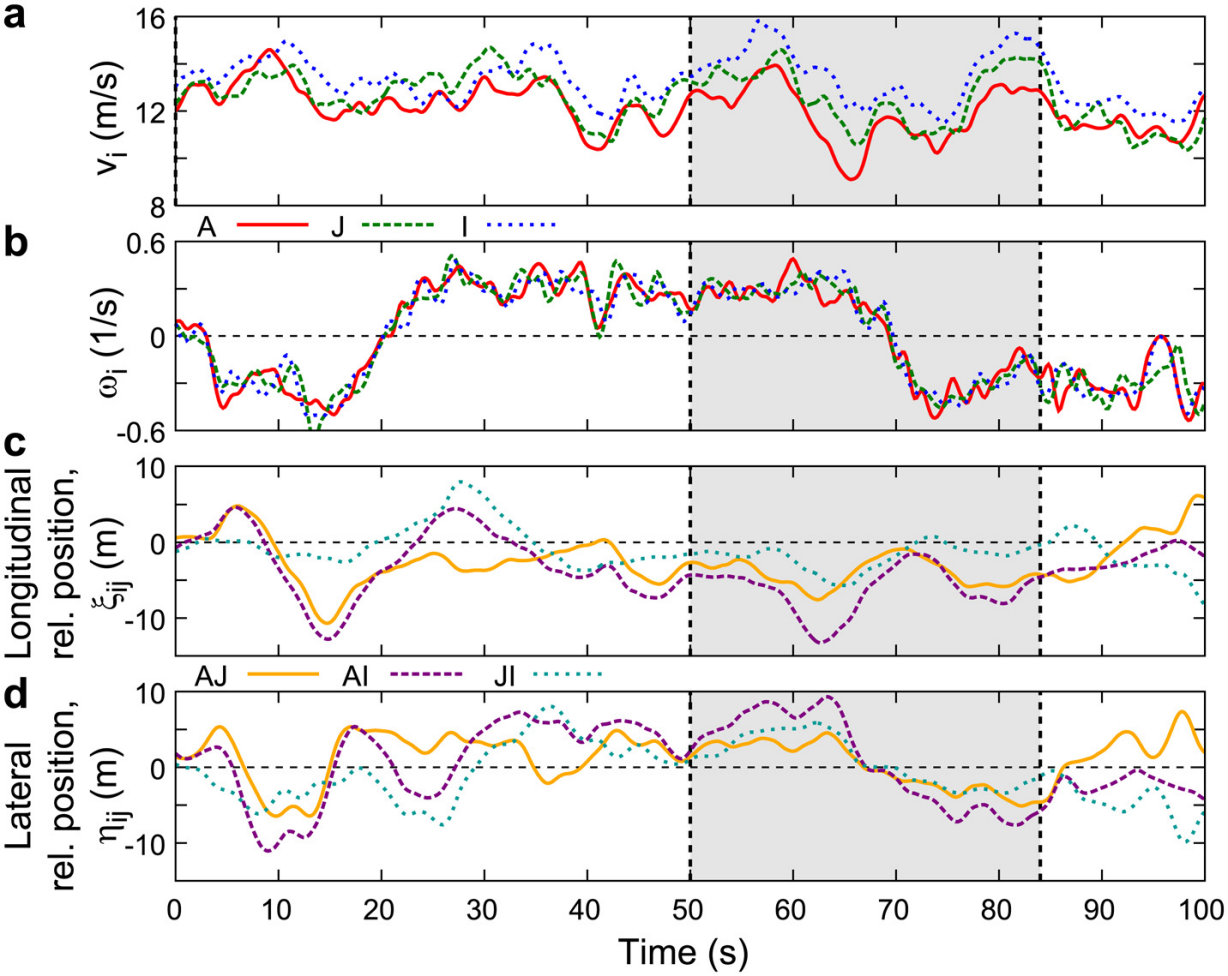
Typical time series of physical quantities characterizing the flights. (a) Velocity; (b) horizontal angular velocity; (c) longitudinal relative position; (d) lateral relative position. In all subplots indices *i* and *j* correspond to individuals A, J and I of Fig 1. The interval from 50 s to 84 s, highlighted in grey, corresponds to the trajectories shown in Fig 1.

Fig 3 shows the time series of the above mentioned physical quantities for the same flight period as in Fig 1. Fig 3b shows that group members fly with similar horizontal angular velocity. Any change in the circling direction is apparent as a change of sign of the angular velocity. Fig 3c and 3d show that the relative positions tend to be maintained for short time scales.

For most of the analysis, we used temporal averaging to filter noise. The symbol 〈⋅〉 is used for temporal averaging. We used *T* = 20 s as averaging time, but we also checked that all results are qualitatively robust using different values of *T* in the range of 10 ∼ 40 s. Fluctuations become larger if *T* is smaller than 20 s, due to changing wind, bias of the measurement system, etc. By setting *T* = 20 s, which is approximately the period of one cycle of the circling trajectories, some of these errors are expected to average out. For example, if the offset error between different GPS devices is constant in one direction or the wind is blowing from one direction, the integral errors during a full circle will be cancelled.

We regarded a bird as being in a landing or take-off state if $|{\vec{r}}_{i}\left(t_{n}\right)-{\vec{r}}_{i}\left(t_{n}-10\;\text{s}\right)|\leq 50\;\text{m}$ or $|{\vec{r}}_{i}\left(t_{n}\right)-{\vec{r}}_{i}\left(t_{n}+10\;\text{s}\right)|\leq 50\;\text{m}$ at time *t*_*n*_. We excluded these periods from all analyses. We also excluded an individual data when the distance to the nearest neighbour was more than 50 m.

To characterise the structure and dynamics of flocks, we regarded flocks as composites of pairs of individuals and focused on the dynamics of the relative positions and angular velocities of each pair.

For the rotational motion analysis we did not perform time averaging, but excluded data points for which i) birds were > 10 m away from the centre of mass; ii) there were fewer than five birds in the flying state; iii) velocity of centre of mass was smaller than 5 (m/s).

## Results

### Characteristic relation between temporal and spatial order

In previous studies [8, 21], a hierarchical leader-follower relationship was defined based on the delay $\tau_{ij}^{c}$ between directional changes of individuals *i* and *j*. In these studies the delay was only considered statistically, as an average for entire flights. Contrarily, in this paper we focus on the instantaneous relationship between pairs during short periods (*T* = 20 s). First, we divide the flight data into intervals of length *T*, then define the leader-follower relationships for all pairs during each interval. To calculate $\tau_{ij}^{c}$, we measure the correlation function of the direction of motion between the *i*-th and the *j*-th individual:

$$C_{ij}(\tau )=\langle \frac{{\vec{v}}_{i}(t)}{\left|{\vec{v}}_{i}(t)\right|}\cdot \frac{{\vec{v}}_{j}(t+\tau )}{\left|{\vec{v}}_{j}(t+\tau )\right|}\rangle ,$$ (1)

where 〈 〉 represents a time average for *T*. We define the delay time $\tau_{ij}^{c}$ as *τ* where *C*_*ij*_ takes its maximum, as in the previous studies. The leader-follower relationship is defined between each pair from the sign of $\tau_{ij}^{c}$, i.e., if $\tau_{ij}^{c}>0$ then *i* is followed by *j* during *T*. During each time interval, we also quantify the relative position of the *j*-th individual in the individual coordinate system of the *i*-th individual (〈*ξ*_*ij*_〉, 〈*η*_*ij*_〉).

In the flight data of hooded gull flocks measured by a stereo camera system [10], the relation between instantaneous temporal and longitudinal spatial order was found to be:

$$\langle \xi_{ij}\rangle =-V\tau_{ij}^{c},$$ (2)

where *V* denotes the average velocity of motion. We investigated this relationship between 〈*ξ*_*ij*_〉 and $\tau_{ij}^{c}$ in all four datasets of pigeons (D1, D2A, D2B, D2C).

**Fig 4:**
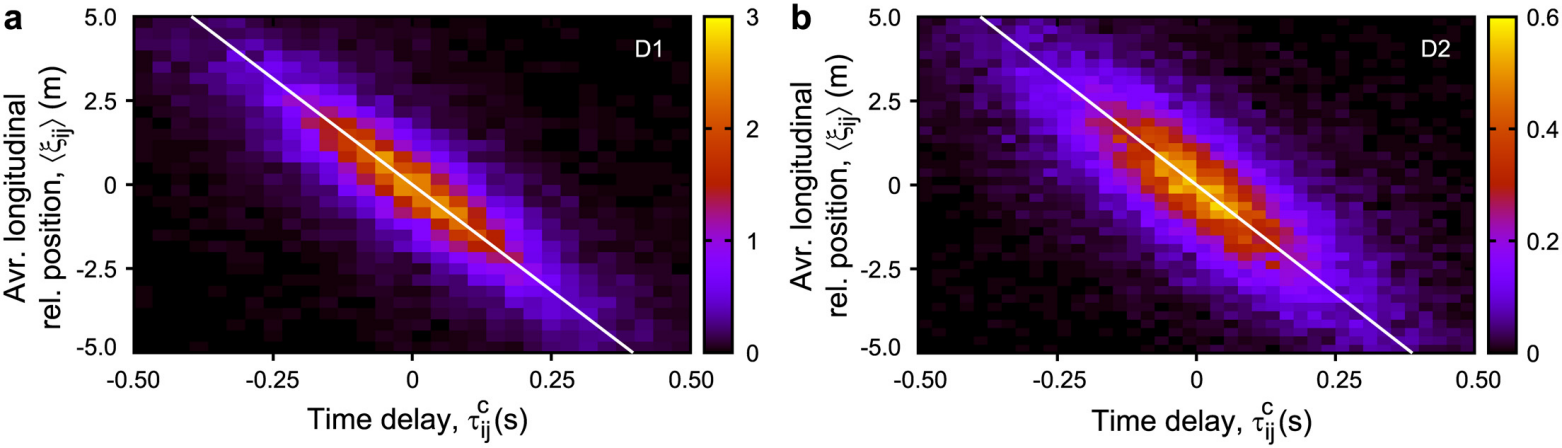
Relationship between temporal and spatial order. Temporal order is represented by the delay of direction change $\tau_{ij}^{c}$, spatial order is represented by the longitudinal relative position 〈*ξ*_*ij*_〉. Distributions are similar for both D1 (a) and D2 (b) datasets and correspond to Eq (2). The white solid line represents the slope corresponding to the average velocity of each dataset. Detailed statistical quantities are given in Table 2.

Fig 4 shows the distribution of 〈*ξ*_*ij*_〉 versus $\tau_{ij}^{c}$ for D1 and D2 (D2A, B, C combined). The white solid line represents the slope −*V*, corresponding to the average velocity of all members in the given time period. This line fits the distributions well, meaning that Eq (2) is valid not only for hooded gull data but also for pigeons (exact parameters of the fitting are summarized in Table 2).

**Table 2:** Statistical quantities of all datasets.

		D1	D2A	D2B	D2C
number of data points, *N*		1.3 × 10^4^	1.3 × 10^4^	0.3 × 10^4^	0.9 × 10^4^
speed, *v* (m/s)	mean	12.4	12.7	11.8	12.8
	S.D.	2.2	3.2	3.7	3.6
angular velocity, ∣*ω*∣ (s^−1^)	mean	0.35	0.33	0.39	0.36
	S.D.	0.18	0.20	0.22	0.21
curvature, ∣*κ*∣ (m^−1^)	mean	0.030	0.028	0.036	0.031
	S.D.	0.018	0.022	0.026	0.023
(−*Vτ* ^*c*^)−(*ξ*) fit	slope	1.16	0.99	1.07	0.98
	Pearson’s *r*	0.77	0.69	0.72	0.69
(*v*_*i*_ cos *θ*_*ij*_−*v*_*j*_)−(*ω*_*i*_ *η*_*ij*_) fit	slope	1.05	1.11	1.05	1.15
	Pearson’s *r*	0.86	0.81	0.79	0.75
(sin *θ*_*ij*_)−(*ω*_*i*_ *ξ*_*ij*_) fit	slope	1.05	1.05	1.07	1.03
	Pearson’s *r*	0.79	0.80	0.80	0.78

**Fig 5:**
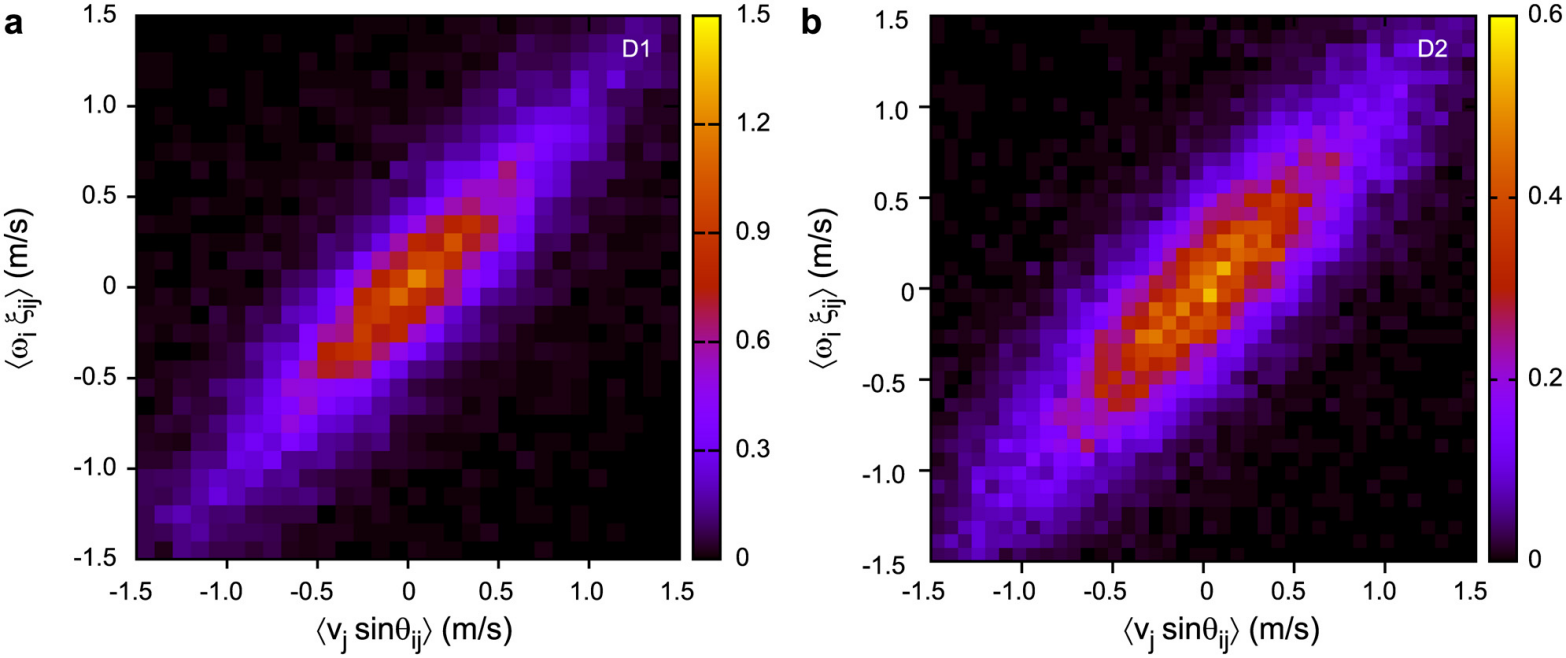
Relationship between 〈*v*_*j*_ sin *θ*_*ij*_〉 and 〈*ω*_*i*_ *ξ*_*ij*_〉. Points on the diagonal represent the case when lateral relative position is maintained. Both D1 (a) and D2 (b) datasets display such an average tendency.

**Fig 6:**
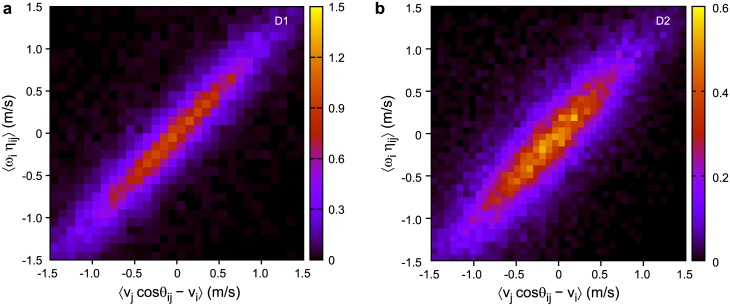
Relationship between 〈*v*_*j*_ cos *θ*_*ij*_ − *v*_*i*_〉 and 〈*ω*_*i*_ *η*_*ij*_〉. Points on the diagonal represent the case when longitudinal relative position is maintained. Both D1 (a) and D2 (b) datasets display such an average tendency.

The first three rows summarize mean and S.D values of some of the primal quantities of the orbits, i.e., speed, absolute angular velocity and absolute curvature. The last three rows contain fitting parameters corresponding to Figs 4, 5 and 6. Fitted slopes are calculated using principal component analysis. Pearson’s *P* of each relation is less than 0.001. *N* is the number of analysed time intervals of length *T*, multiplied by the number of pairs in each interval.

Note that according to Eq (2), the followers tend to be located behind their leaders. A similar relation was found in the previous study [8] quantifying average behaviour, and our analysis supports it quantitatively when considering smaller segments (circles) separately.

Eq (2) connects the temporal order of direction change to the longitudinal spatial order of individuals within the flock. Next, we give a kinematic interpretation of Eq (2). Let us consider the time derivatives of the longitudinal (*ξ*_*ij*_) and lateral (*η*_*ij*_) relative positions. If we assume that the motion is horizontal (as indicated by Fig 1 and S2 Fig), ${\dot{\xi }}_{ij}$ and ${\dot{\eta }}_{ij}$ can be written as follows:

$${\dot{\xi }}_{ij}=v_{j}\cos\theta_{ij}-v_{i}-\omega_{i}\eta_{ij}$$ (3)

$${\dot{\eta }}_{ij}=-v_{j}\sin\theta_{ij}+\omega_{i}\xi_{ij},$$ (4)

where *θ*_*ij*_ ≡ *θ*_*j*_ − *θ*_*i*_ (see S1 Text).

Let us also assume that individuals fly more or less in parallel, i.e., ∣*θ*_*ij*_∣ ≪ 1. Then Eqs (3) and (4) can be approximated as:

$${\dot{\xi }}_{ij}\approx v_{j}-v_{i}-\omega_{i}\eta_{ij}$$ (5)

$${\dot{\eta }}_{ij}\approx -\omega_{i}\left(v_{j}\frac{\theta_{ij}}{\omega_{i}}-\xi_{ij}\right).$$ (6)

In Eq (6), *θ*_*ij*_/*ω*_*i*_ represents a characteristic time scale determined by the difference in angle between the pair and the angular velocity. This time scale recalls $\tau_{ij}^{c}$, the time delay between individuals *i* and *j* changing direction, introduced at the beginning of this section. Therefore, Eq (2) is consistent with ${\dot{\eta }}_{ij}=0$ under the assumption:

$$\frac{\theta_{ij}}{\omega_{i}}=-\tau_{ij}^{c}.$$ (7)

In other words, Eqs (2) and (7) are necessary conditions for the individuals to maintain their relative lateral positions during turning flight, and Eq (2) is a meaningful relation not only for longitudinal relative position, but also for maintaining lateral relative position.

To check how much the lateral relative positions were actually maintained, we analyse the right side of Eq (4). Fig 5 shows the distribution of 〈*v*_*j*_ sin *θ*_*ij*_〉 versus 〈*ω*_*i*_ *ξ*_*ij*_〉 for datasets D1 and D2. The distributions have a positive correlation with a slope close to unity (exact slope and Pearson’s *r* are given in Table 2), meaning that the right side of Eq (4) vanishes more or less, thus the tendency is that lateral relative positions are maintained.

Note that the above results only represent the trend on an average. The fluctuations from the diagonal line in Fig 5 mean that lateral relative positions do change from time to time. Detailed analysis in the next subsection shows that the magnitude of this tendency depends on the flight state of the flock.

Similar analysis can be applied to the longitudinal relative position (*ξ*_*ij*_), using the right side of Eq (4). Fig 6 shows the distribution of 〈*v*_*j*_ cos *θ*_*ij*_ − *v*_*i*_〉 versus 〈*ω*_*i*_ *η*_*ij*_〉 for datasets D1 and D2. Positive correlation with unit slope is observed again, thus the longitudinal relative positions are also maintained in pigeon flocks on average. This result together with Eq (2) can be interpreted as the existence of a hierarchical leadership network in the flock.

Note that the tendency of individuals to maintain their relative positions within the flock seems to be weaker in the D2 datasets than in D1. There are several differences between the two experiments as described in Table 2. Further, more systematic measurements will help us clarify the main differentiating factors behind these behavioural differences.

For individuals to maintain their lateral relative positions perfectly, they must fly on parallel orbits. This is a rather strong constraint to the flight behaviour, because parallel orbits require different flight speeds and different turning radii. There must be cases in which this property breaks down, which correspond to the deviation from the diagonal line in Fig 5. In the next subsection we analyse further when the parallel, P type turning are maintained.

### Rotational motion in the flock

The goal of this subsection is to determine under what conditions pigeons can maintain their relative positions by taking parallel-path trajectories, and if they are unable to satisfy this ideal case, to determine how much their paths follow equal-radius type turning.

**Fig 7:**
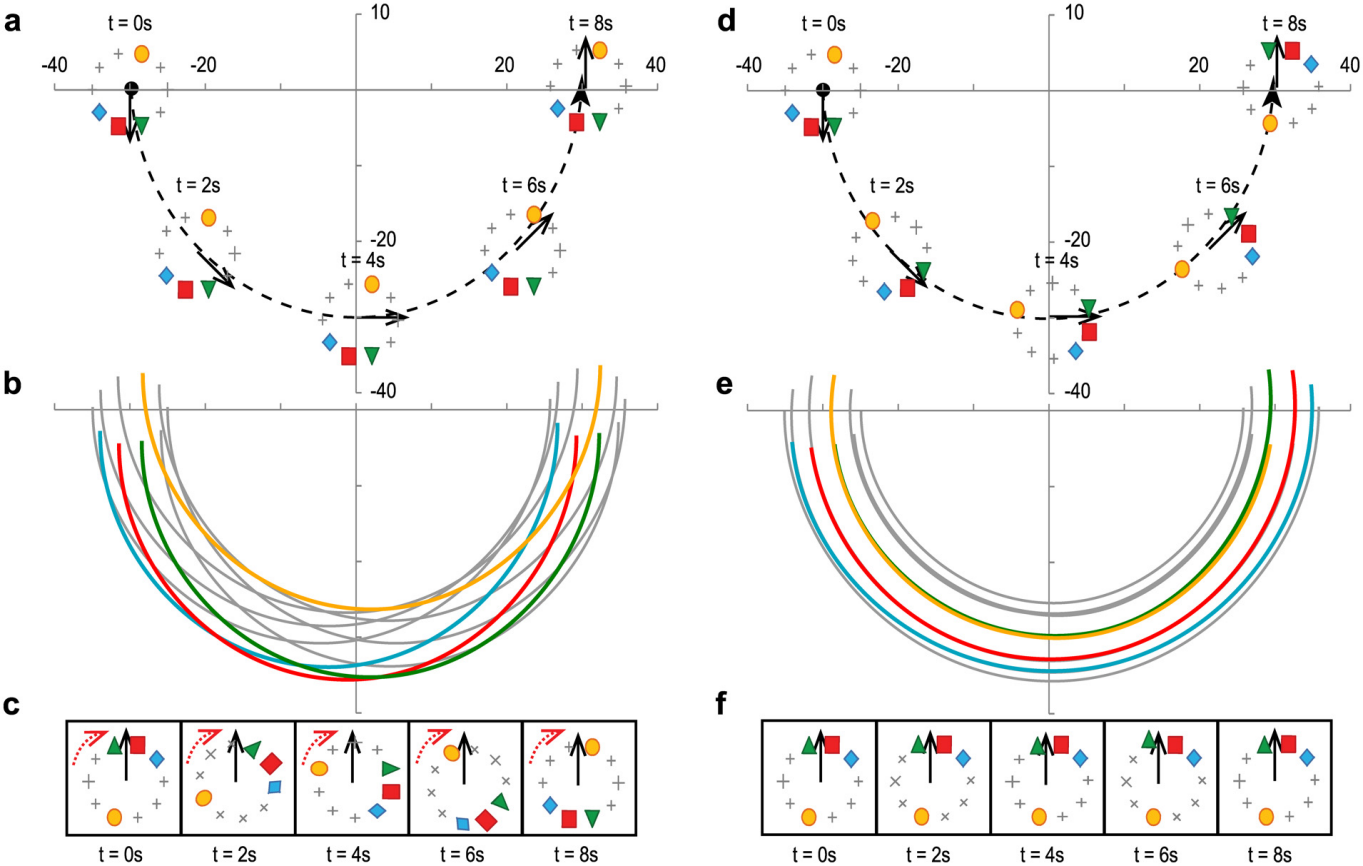
Illustration of the equal radii (a)-(c) and parallel path (d)-(f) turning cases. Panels show half-circle long trajectories of individuals belonging to idealistic flocks flying on a radius of 30 m. The internal structure of the flock was modelled by distributing 10 individuals evenly along a circle with a radius of 5 m. (a) During E type turning the orientation of the internal structure is fixed to a global coordinate system. The trajectories of individuals draw circles with a common radius as shown in (b). In the flock’s co-moving coordinate system (shown in (c)) the flock is rotating, as indicated by red arrows. The rotation is in the opposite direction compared to the turning, and individuals at front end up at the back of the flock after a half-circle turn. (d) During P type turning the internal structure of the flock is not rotating in the co-moving coordinate system. And individuals fly along concentric circles as shown in (e). In this case, individuals keeps both their longitudinal and lateral relative positions as in (f).

First, we exemplify these two idealistic turnings by artificial bird flocks in Fig 7. In E type turning (Fig 7a–7c), all individuals fly with a common constant speed and turn with a common curvature radius simultaneously with different centres of rotation as in Fig 7b. Therefore, the orientation of the internal structure is fixed to a global coordinate system. In the flock’s co-moving coordinate system (shown in Fig 7c) the flock is rotating. On the other hand, in P type turning (Fig 7d–7f), all individuals fly with a common constant angular velocity and turn around the common centre of rotation with different speed. Therefore, the orientation of the internal structure is fixed in the flock’s co-moving coordinate system and the the orbits do not intersect as in Fig 7f and 7e.

**Fig 8:**
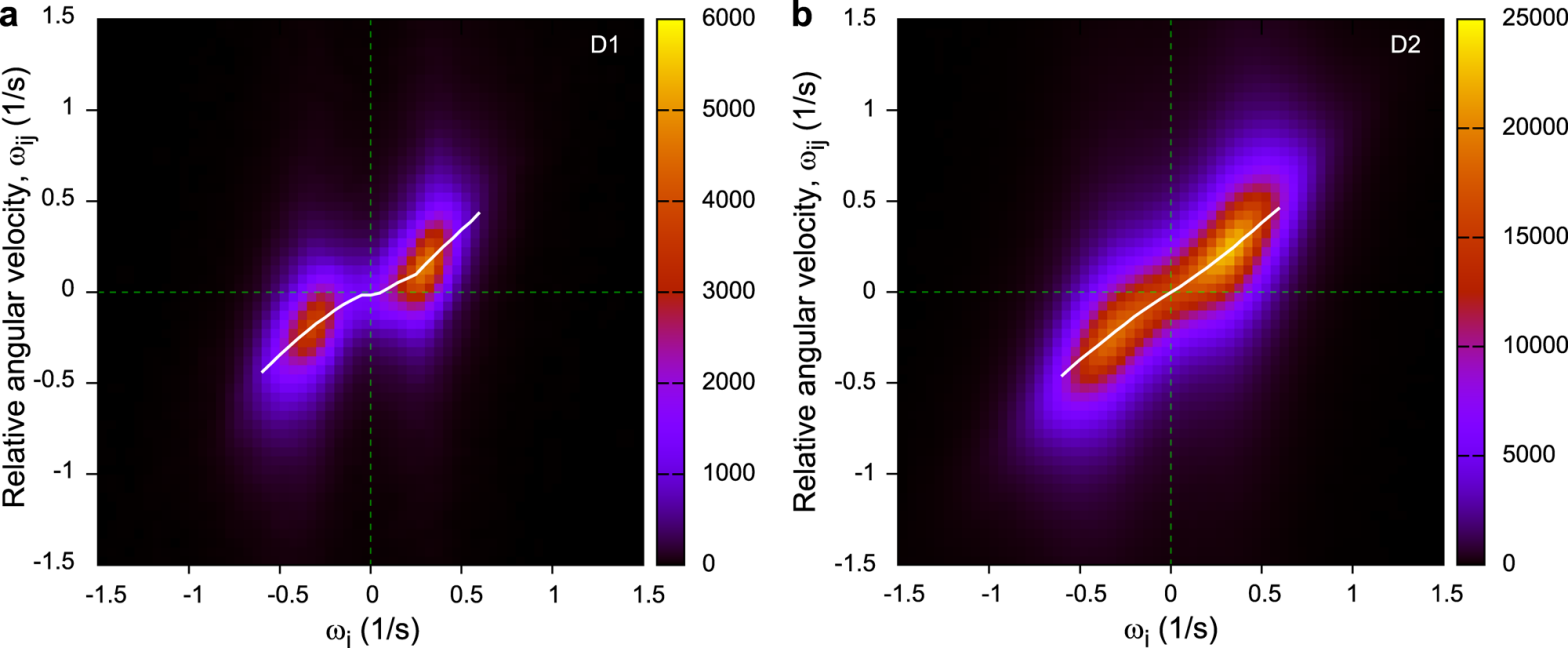
Analysis of the internal rotation during turning. The white solid lines represent the averaged of *ω*_*ij*_ for each *ω*_*i*_. P type turning would result in the line *ω*_*ij*_ = 0, while E type turning would result in *ω*_*ij*_ = *ω*_*i*_. Both D1 (a) and D2 (b) datasets show characteristics of these two idealised cases, with E type turning more prevalent at high angular velocities. Plot contains summarized data for all pairs. Note that D1 data contains mostly circles and fewer straight trajectories, i.e., it is more regular, that is why the middle of the distribution is missing.

For the analysis, we plot the distribution of angular velocities, namely, *ω*_*i*_ versus *ω*_*ij*_, where $\omega_{ij}={\dot{\psi }}_{ij}$ is the relative angular velocity of individual *j* in the coordinate system of individual *i* (Fig 8). Note that this time we do not apply the previously used time averaging since it would cancel out the effects we are trying to investigate.

On Fig 8, perfect parallel path (P type) turning would result in a straight line with slope zero, since *ω*_*ij*_ would always remain zero during turning. On the other hand, perfect equal-radius (E type) turning would result in a line with a slope of one, because during E type rotation individuals also rotate relative to each other within the flock (see also Fig 7 and S3 Fig). Note that the positive direction of the global *θ*_*i*_ is opposite to the positive direction of the local *ψ*_*ij*_.

In the real distributions, we see characteristics of both idealistic turning methods, with more P type turning at low angular velocities and more E type turning at high angular velocities in Fig 8a. The white solid lines in Fig 8 are the average of *ω*_*ij*_ for each *ω*_*i*_. This is consistent with the fact that during turns with high angular velocity, maintaining parallel paths is more difficult if changing the speed of flight is costly.

Note that GPS position offset errors can significantly modify the *ω*_*i*_-*ω*_*ij*_ distribution and push it towards the E type outlook. See S3 Fig for a detailed analysis of this effect.

The second method we used to differentiate between P and E type turning was as follows. We took the longitudinal relative position *ξ*_*ij*_ and searched for $\xi_{ij}^{\pi }$ taken from the most recent time instant in the last 40 s when the orbiting phase *θ*_*i*_ of individual *i* was opposite, i.e., a half-circle backwards (with ±*π*/36 tolerance). If such a time instant could not be found, i.e. birds were not actively circling, data points were neglected. If we plot the distribution of *ξ*_*ij*_ versus $\xi_{ij}^{\pi }$, the two turning methods would once again give us different distributions. P type turning would result in a single dot along the diagonal, with its location representing the average relative longitudinal position between individuals *i* and *j*. E type turning would result in a straight line with a slope of minus one, because during a half-circle turn the relative positions of the individuals are exchanged (see also Fig 7 and S3 Fig).

**Fig 9:**
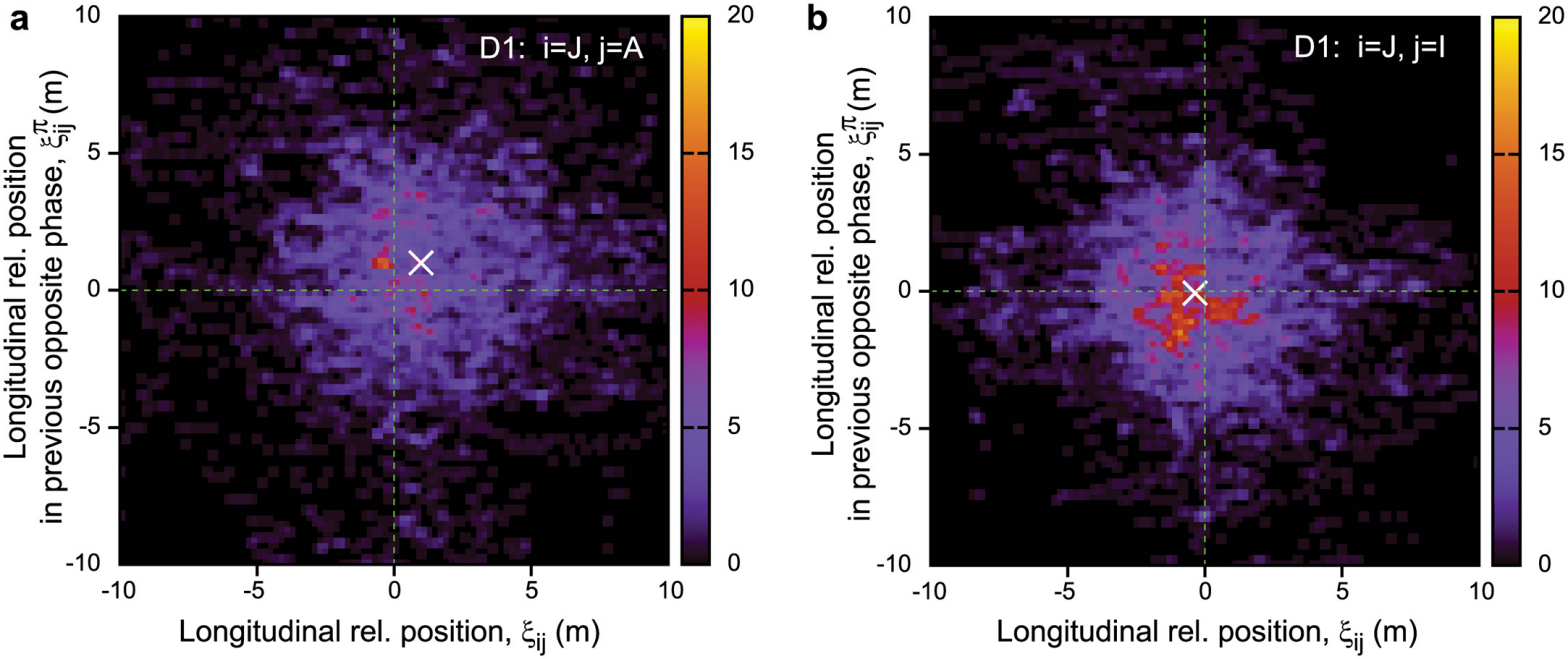
Change of longitudinal relative position during a half-circle. Distribution of the instantaneous longitudinal relative position as a function of the same quantity a half-circle earlier. Representative examples are taken from dataset D1 (same birds that are shown on Figs 1 and 3). A is known to be a leader bird; J and I are at the middle of the hierarchy. Individual A clearly tends to be in front of J (*ξ*_*JA*_ is positive on average), while J and I don’t have much difference in their longitudinal positions. The average of the distribution is represented with a white cross. The offset of the cross corresponds to the average longitudinal position offset between the given birds. Perfect P type turning would be a single point along the diagonal, while perfect E type turning would be a line with slope −1.

Examples of representative pairs with summarized data from all flights in D1 are presented in Fig 9. Data from D2 are very similar (see S3 Fig for more details). The real scenario is once again far from any of the idealistic cases. The centre of the distribution once again resembles P type turning, with definite positional differences between leader and follower birds. However the distribution has large standard deviation, and here there is no significant resemblance to the idealistic E type orbiting.

In summary, birds never take ideal turns. On average they tend to keep fixed positions within the flock, but the flock is much more dynamic than a rigid body. Instantaneous positions change frequently and small radius turns (turns with large angular velocity) seem to give rise to the E type turning method, too.

### Relation between leaders and followers

In this subsection we restrict our analysis to pairwise leader-follower interactions only. The leader or follower role of a given individual in each pair was assigned based on the delay calculated from the direction correlation function over T time duration. Hereafter, indices “*l*” and “*f*” denote leaders and followers, respectively. For example, *v*_*l*_ is the velocity of a leader and *ξ*_*lf*_ is the longitudinal relative position of a follower in a leader’s individual coordinate system. The following results are pooled for all leader-follower pairs in all releases.

**Fig 10:**
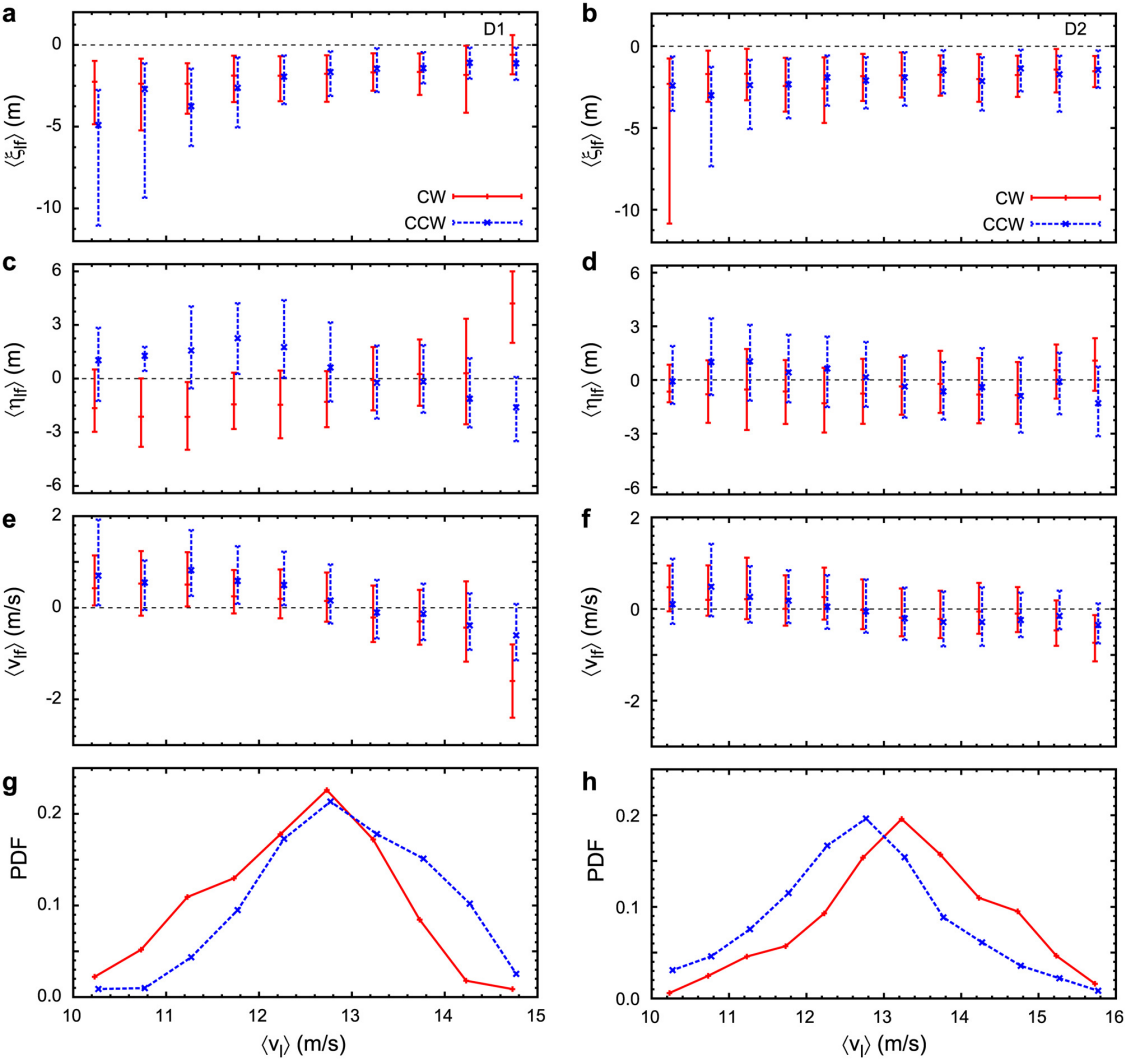
Relative position and velocity of followers in CW and CCW circling, expressed as a function of the leader’s velocity. Left and right columns are of datasets D1 and D2, respectively. (a),(b): longitudinal position of follower relative to leader; (c),(d): lateral position of follower relative to leader; (e),(f): velocity of follower relative to leader; (g),(h): distribution of leader velocity. For better visibility, each graph is shifted 0.02 left for CW and 0.02 right for CCW, respectively. Symbols and error bars indicate the median and the area between the lower and higher quartile points from (a) to (f).

Fig 10 shows different quantities as a function of the leader’s velocity. Considering the longitudinal relative position (Fig 10a), followers tend to be located behind their leaders, irrespective of the leader’s velocity or turning direction. This is consistent with Fig 4, i.e., leaders change direction earlier and they are also located at the front.

On the other hand, the follower’s lateral relative position (Fig 10c) and relative velocity (Fig 10e) is dependent on the leader’s velocity. At low leader velocity (*v*_*l*_ < 13 m/s), followers fly faster (*v*_*lf*_ > 0) but take the outer—and longer—orbits (*η*_*ij*_ > 0 for CCW and *η*_*ij*_ < 0 for CW turns), to remain behind leaders. Contrarily, at high leader velocity (*v*_*l*_ > 14 m/s), followers are slower but take the inward—and shorter—orbits, to keep up with leaders.

Fig 10g shows the distribution of leader velocity. It shows that *v*_*l*_ ≲ 11 m/s and *v*_l_ ≳ 14 m/s are somewhat “rare” events; therefore, error bars in Fig 10a, 10c and 10e in this range are relatively longer.

Also note that these results on the lateral relative position and the relative velocity of followers are more specific to D1 (Fig 10a, 10c, 10e, 10g—left panels) and are somewhat less obvious for the D2 (Fig 10b, 10d, 10f, 10h—right panels) datasets.

A typical example of the described behaviour is shown in Figs 1 and 3. Bird A typically flies slower than I and J and in the meantime it takes the shorter, inward orbit in both CW and CCW turns, which results in the reconfiguration of lateral distances when circling direction changes. Note that some individuals change their positions more frequently, which correspond to the out-of-line distribution in Figs 5 and 6.

## Discussion

In this paper we revealed new aspects of the relative motion and the turning method of pigeons in circling group flights. In the literature, there are two kinds of ideal orbits: equal radii paths and parallel paths. The former causes internal rotational and translational motion while the latter maintains relative positions without internal rotation. Interestingly, according to the experimental data, actual orbits represent a mixture of these ideal cases.

During P type turning, the orbits do not intersect each other, and birds keep their flight “lanes”. This behaviour is useful for flying objects (migrating birds or even fixed-wing airplane flocks) to minimize energy consumption during flight. Namely, there are two vortex lines emitted backwards from the ends of wings. The consecutive bird/aircraft can benefit from the upwash of the rotating vortex if it is located just outside the vortex line. This behaviour was already found in several species [7, 10]. Note that P type motion or turning is also exhibited in dense flocks of birds [20] and fish schools [18, 19], where close neighbours act as a physical constraint that simply does not allow for low polarization, i.e. crossing orbits of the E type turning.

However, it was found that for pigeons flying in such a tight flock actually comes with aerodynamic costs rather than benefits [9]. We also found that if the angular velocity is too large (or the curvature radius is too small), it is difficult to maintain relative positions within the flock. The time derivative of the lateral relative position is proportional to the angular velocity as shown in Eq (6). In this case, their relative position in the flock’s co-moving coordinate system rotates each other as in the E type turning. Pomeroy also reported that airborne rock dove flocks do not form P type turning [22]. This could also be attributed to a tight turning scenario. The estimated angular velocity according to Fig 4 in [22] is at least twice as high as the typical values present in our datasets. In summary, we assume that actual flocks change their strategy depending on the situation/environment and the ability and will of the members.

Next, we comment on the differences between datasets D1 and D2. There are some observational and environmental differences, and from the trajectory analysis it is obvious that the regularity of the turning flights is different (see electronic supplementary material, S1 Fig). However, we do not understand what the key differences are that affect the flight behaviour most. Causes may include level of prior training or particular landmarks near the turning flights. This is an open problem. Datasets D2A, D2B and D2C have different distribution functions of *ω*_*i*_, whereas the distribution functions of $\dot{\omega_{i}}$ resemble each other well. The distribution function of $\dot{\omega_{i}}$ may grasp an important property of the flight orbits.

Furthermore, errors in the three D2 datasets seem to be larger than those of D1. This might be caused by the fact that circling direction in the D2 datasets changes more frequently than in D1. In this paper, we averaged physical quantities for a period of approximately one cycle of the trajectory to cancel some errors, such as wind or the difference between GPS devices. Note that we can estimate wind velocity from the circling flights, but doing so does not alter results significantly (see S2 Text). The error magnitude may increase when we analyse behaviour over a shorter time than one circling period. This ambiguity could be resolved in the future by more precise GPS devices or other analysis methods.

Finally, we comment on the velocity difference between leaders and followers. When leaders fly more slowly, followers tend to fly faster and on a more outward orbit. It may be that leaders have the ability to slow down in the air, which increases the risk of stalling [23]. Note that in the analysis of Pettit et al., on pigeons belonging to the same loft as in the D2 datasets, pigeons with higher solo flight speed tend to lead their colleagues in flock flights [24]. We think that this discrepancy is due to their different flight states. Data analysed by Pettit are for *homing* flights, i.e., when pigeons fly back from a distant place to their loft. On the other hand, data analysed in this article consists of *free* flights, i.e., when birds fly near their loft spontaneously. It is known that these two flight states have different average velocities (S4 (a) Fig). We also checked the relationship between the average flight speeds during free and homing flights for each individual, and slower individuals in free flights can fly faster during homing flights (S4 (b) Fig). Therefore, our result for the relationship between speed and leadership does not conflict with that of Pettit.

## Supporting Information

S1 FigDistributions of the (a) angular velocity, *ω*, and the (b) angular acceleration, $\dot{\omega }$, both in the horizontal plane for all pigeons and observation time.Each distribution function corresponds to a different dataset. All distribution functions of *ω* show two peaks around ±0.3 ∼ 0.4 rad/s, however, the distribution of dataset D1 is more symmetric with respect to *ω* = 0, and peaks in D1 are sharper than in D2. Each distribution function of $\dot{\omega }$ exhibits a single peak structure at $\dot{\omega_{i}}=0$, however, the variance of the distribution is smaller in D1 than in D2. Thus, the flocks of dataset D1 fly more regularly than the flocks of the D2 datasets.(EPS)

S2 FigDistributions of the velocity components (a) and the angular velocity components (b) of the vertical and horizontal directions for all individual flights in the case of dataset D1.
*v*_*xy*_ indicates the horizontal velocity, $v_{xy}=\sqrt{v_{x}^{2}+v_{y}^{2}}$. The average values and the standard deviations of *v*_*xy*_ and ∣*v*_*z*_∣ are 12.9 ± 1.8 m/s and 0.01 ± 0.7 m/s, respectively. *ω* and *ω* ^⊥^ indicate the vertical and the horizontal components of the angular velocity, respectively. Namely, $\omega =\dot{\theta }$ with $\theta =\tan^{-1}(\frac{v_{y}}{v_{x}})$ and $\omega^{⊥}=\dot{\phi }$ with $\phi \equiv \tan^{-1}(\frac{v_{xy}}{v_{z}})$. The average values and the standard deviations of ∣*ω*∣ and ∣*ω* ^⊥^∣ are 0.35 ± 0.17 rad/s and 0.01 ± 0.01 rad/s, respectively. See main text and S1 Text.(EPS)

S3 Fig Comparison of the datasets to idealistic turning cases. Trajectories were generated for E and P type cases as shown in Fig 7. Other parameters of the simulation: duration = 700 s, speed of the flock’s centre of mass = 12 m/s, radius increases linearly from 30 m to 100 m, turning is exhibited only in CCW direction. Panels from the left to right show statistics for E type (left), P type (middle left) simulated datasets and D1 (middle right) and D2 (right) experimental datasets. (a) Internal rotation as shown in Fig 8. Internal rotation of E type turning is shown by the diagonal line (left panel). During P type turning there is no internal rotation, indicated by the *ω*_*ij*_ = 0 (middle left panel). (b) Panel shows the same statistics as panel (a) with added artificial noise. To estimate the effect of time-correlated systematic GPS noise, each trajectory was shifted artificially by 5 m to a random direction for each individual in each release. When positions of individuals in the internal structure are fixed to global coordinate system, relative internal rotation appears in the co-moving coordinate system. Distributions shift towards the diagonal to resemble more to the E type turning. (c) Change of the longitudinal relative position during a half-circle turn in the simulated and the experimental datasets as shown in Fig 9 for selected pairs. Inset shows distribution for another pair (except for E type turning where all distributions are identical). During E type turning individuals switch their internal longitudinal positions but the average (denoted by the white X mark) remains in the origin. P type turning results in a very narrow distribution (equals to the average position) along the diagonal. This location is characteristic to the individual. (d) Same statistics as on panel (c) with added artificial noise. Distributions become more elongated in the direction of the −1 diagonal, but this type of bias does not effect the position of the average of the distributions considerably. (e) The average of the distribution for all possible pairs for the original/unbiased datasets (shown by red X marks) and for the artificially biased cases (shown by blue + marks). The experimental datasets have the characteristics of the P type turning.
(TIF)

S4 Fig (a) Distribution of velocity magnitude for free and homing flights and (b) the relation between average velocity of free and homing flights for each individual. The average velocity for homing flights is higher than that for free flights. The average velocity of each pigeon in free and homing flights has a negative correlation except for pigeons G and L. The negative correlation means that a pigeon flying slowly in free flights flies fast in homing flights. In our analysis, pigeons flying slowly in free flights tend to lead other pigeons, whereas in homing flights pigeons flying faster tend to lead other pigeons [24]. Therefore, it is understood that leading pigeons have wider speed range than followers. The homing flight data of pigeons G and L have a bimodal velocity distribution and one peak value is almost the same as the peak value of free flights. Thus, their average homing flight velocity may be underestimated, due to the inclusion of free flights in homing flight data.
(EPS)

S1 Video Animations showing GPS data of a 70 s segment of free flight by 10 pigeons from dataset D1 (2.5x real speed). Local time is shown in the bottom left corner. Individuals are coloured according to their ranks in the hierarchy, determined by pairwise directional correlation delay times for the whole flight. Colors near the red end of the spectrum indicate higher leadership on average. Inset shows the trajectories in coordinates fixed to the ground while the main plot shows the trajectories in a co-moving coordinate system. Grid size is 5 m.
(AVI)

S2 Video Animations showing GPS data of a 70 s segment of free flight by 10 pigeons from dataset D2A (2.5x real speed). Local time is shown in the bottom left corner. Individuals are coloured according to their ranks in the hierarchy, determined by pairwise directional correlation delay times for the whole flight. Colors near the red end of the spectrum indicate higher leadership on average. Inset shows the trajectories in coordinates fixed to the ground while the main plot shows the trajectories in a comoving coordinate system. Grid size is 5 m.
(AVI)

S1 Text Relative velocity in individual coordinates. (PDF)

S2 Text Estimation of wind. (PDF)
