# Supplementary figures and images for: Coordinated Behaviour in Pigeon Flocks
Source: PLoS One. 2015 Oct 20;10(10):e0140558. doi: 10.1371/journal.pone.0140558 (PMC4618473; doi:10.1371/journal.pone.0140558)

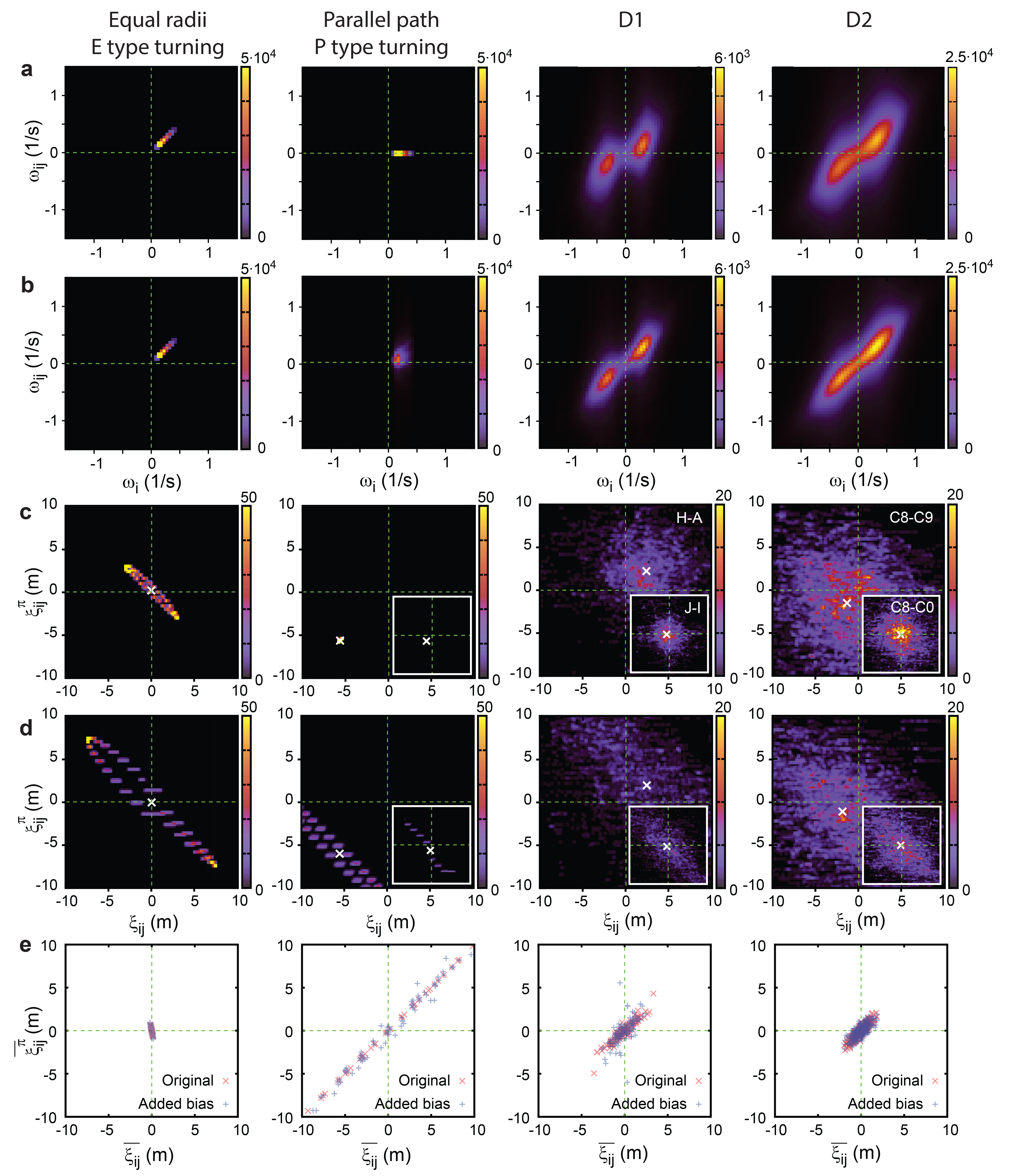

Supplement: S3 Fig — Trajectories were generated for E and P type cases as shown in Fig 7. Other parameters of the simulation: duration = 700 s, speed of the flock’s centre of mass = 12 m/s, radius increases linearly from 30 m to 100 m, turning is exhibited only in CCW direction. Panels from the left to right show statistics for E type (left), P type (middle left) simulated datasets and D1 (middle right) and D2 (right) experimental datasets. (a) Internal rotation as shown in Fig 8. Internal rotation of E type turning is shown by the diagonal line (left panel). During P type turning there is no internal rotation, indicated by the ω ij = 0 (middle left panel). (b) Panel shows the same statistics as panel (a) with added artificial noise. To estimate the effect of time-correlated systematic GPS noise, each trajectory was shifted artificially by 5 m to a random direction for each individual in each release. When positions of individuals in the internal structure are fixed to global coordinate system, relative internal rotation appears in the co-moving coordinate system. Distributions shift towards the diagonal to resemble more to the E type turning. (c) Change of the longitudinal relative position during a half-circle turn in the simulated and the experimental datasets as shown in Fig 9 for selected pairs. Inset shows distribution for another pair (except for E type turning where all distributions are identical). During E type turning individuals switch their internal longitudinal positions but the average (denoted by the white X mark) remains in the origin. P type turning results in a very narrow distribution (equals to the average position) along the diagonal. This location is characteristic to the individual. (d) Same statistics as on panel (c) with added artificial noise. Distributions become more elongated in the direction of the −1 diagonal, but this type of bias does not effect the position of the average of the distributions considerably. (e) The average of the distribution for all p [file pone.0140558.s003.tif]
