# Supplementary material for: Coordinated Behaviour in Pigeon Flocks
Source: PLoS One. 2015 Oct 20;10(10):e0140558. doi: 10.1371/journal.pone.0140558 (PMC4618473; doi:10.1371/journal.pone.0140558)
Supplement: S1 Text — (PDF) [file pone.0140558.s007.pdf]

S1 Text. Relative velocity in individual coordinates

Coordinated behaviour in pigeon flocks

M. Yomosa, T. Mizuguchi, G. Vásárhelyi ans M. Nagy

Let the three-dimensional position and velocity of the i-th individual be $\vec{r}_i = (r_i^x, r_i^y, r_i^z)$ and $\vec{v}_i = (v_i^x, v_i^y, v_i^z)$ expressed in earth-frame coordinates fixed to the ground. Using these quantities, we define the unit vectors of the individual coordinate system of the i-th individual as follows:

$$\hat{\boldsymbol{e}}_i^\xi = \frac{\vec{v}_i}{v_i}, \tag{S1.1}$$

$$\hat{\boldsymbol{e}}_i^\eta = -\frac{\vec{v}_i \times \vec{g}}{|\vec{v}_i \times \vec{g}|}, \tag{S1.2}$$

$$\hat{\boldsymbol{e}}_i^\zeta = \hat{\boldsymbol{e}}_i^\xi \times \hat{\boldsymbol{e}}_i^\eta, \tag{S1.3}$$

where $\vec{g}$ denotes the unit vector in the direction of gravity and $v_i = |\vec{v}_i|$. The relative position of the j-th individual in the i-th individual coordinate system can be expressed as follows:

$$\xi_{ij} = (\vec{r}_j - \vec{r}_i) \cdot \hat{\boldsymbol{e}}_i^\xi, \tag{S1.4}$$

$$\eta_{ij} = (\vec{r}_j - \vec{r}_i) \cdot \hat{\boldsymbol{e}}_i^\eta, \tag{S1.5}$$

$$\zeta_{ij} = (\vec{r}_j - \vec{r}_i) \cdot \hat{\boldsymbol{e}}_i^\zeta. \tag{S1.6}$$

The time derivatives of the relative position can be expressed as follows:

$$\begin{aligned}
\dot{\xi}_{ij} &= \frac{d}{dt}\left\{(\vec{r}_j - \vec{r}_i) \cdot \hat{\boldsymbol{e}}_i^\xi\right\} \\
&= (\vec{v}_j - \vec{v}_i) \cdot \hat{\boldsymbol{e}}_i^\xi + (\vec{r}_j - \vec{r}_i) \cdot (-\omega_i \cos\phi_i \hat{\boldsymbol{e}}_i^\eta - \omega_i^\perp \hat{\boldsymbol{e}}_i^\zeta) \\
&= v_j \cos\theta_{ij} - v_i - \omega_i \eta_{ij} \cos\phi_i - \omega_i^\perp \zeta_{ij}, \qquad \text{(S1.7)} \\
\dot{\eta}_{ij} &= \frac{d}{dt}\{(\vec{r}_j - \vec{r}_i) \cdot \hat{\boldsymbol{e}}_i^\eta\} \\
&= (\vec{v}_j - \vec{v}_i) \cdot \hat{\boldsymbol{e}}_i^\eta + (\vec{r}_j - \vec{r}_i) \cdot (\omega_i \cos\phi_i \hat{\boldsymbol{e}}_i^\xi + \omega_i \sin\phi_i \hat{\boldsymbol{e}}_i^\zeta) \\
&= -v_j \sin\theta_{ij} + \omega_i \xi_{ij} \cos\phi_i + \omega_i \zeta_{ij} \sin\phi_i, \qquad \text{(S1.8)} \\
\dot{\zeta}_{ij} &= \frac{d}{dt}\left\{(\vec{r}_j - \vec{r}_i) \cdot \hat{\boldsymbol{e}}_i^\zeta\right\} \\
&= (\vec{v}_j - \vec{v}_i) \cdot \hat{\boldsymbol{e}}_i^\zeta + (\vec{r}_j - \vec{r}_i) \cdot (\phi_i \hat{\boldsymbol{e}}_i^\xi - \omega_i \sin\phi_i \hat{\boldsymbol{e}}_i^\eta) \\
&= -v_j \sin\phi_{ij} + \omega_i^\perp \xi_{ij} - \omega_i \eta_{ij} \sin\phi_i, \qquad \text{(S1.9)}
\end{aligned}$$

where $\theta_i = \tan^{-1}(v_i^y/v_i^x)$, $\theta_{ij} = \theta_j - \theta_i$, $\omega_i = \dot{\theta}_i$, $\phi_i = \tan^{-1}\left(v_i^z/\sqrt{v_i^{x\,2} + v_i^{y\,2}}\right)$, $\phi_{ij} = \phi_j - \phi_i$, $\omega_i^{\perp} = \dot{\phi}_i$.

If $\hat{\boldsymbol{e}}_i^{\zeta} = \vec{g}$, that is, $\hat{\boldsymbol{e}}_i^{\xi}$ and $\hat{\boldsymbol{e}}_i^{\eta}$ are included in the horizontal plane, the time derivatives of the relative position can be expressed as follows:

$$\dot{\xi}_{ij} = v_j \cos\theta_{ij} - v_i - \omega_i \eta_{ij}, \tag{S1.10}$$

$$\dot{\eta}_{ij} = -v_j \sin\theta_{ij} + \omega_i \xi_{ij}, \tag{S1.11}$$

$$\dot{\zeta}_{ij} = 0. \tag{S1.12}$$

Note that we can start from the moving coordinates based on the Frenet-Serret frame. In this case, the binormal vector is almost perpendicular to the horizontal plane and the torsion is close to zero for the pigeons' typical orbits. Furthermore, ξ_{ij} and η_{ij} are the tangential and the normal directional components of the relative vector.
