# Supplementary material for: Coordinated Behaviour in Pigeon Flocks
Source: PLoS One. 2015 Oct 20;10(10):e0140558. doi: 10.1371/journal.pone.0140558 (PMC4618473; doi:10.1371/journal.pone.0140558)
Supplement: S2 Text — (PDF) [file pone.0140558.s008.pdf]

S2 Text. Estimation of wind

Coordinated behaviour in pigeon flocks

M. Yomosa, T. Mizuguchi, G. Vásárhelyi and M. Nagy

The trajectory data obtained from a GPS device or a stereo camera system are relative to the particular ground point, therefore, the velocity calculated from these data is the "ground velocity", i.e., a velocity relative to the ground. However, the "air velocity", a velocity relative to the air, may be important in considering with the flight dynamics. The ground velocity $\vec{v}$ is the sum of the air velocity $\vec{v}^a$ and the wind velocity $\vec{w}$:

$$\vec{v} = \vec{v}^a + \vec{w}. \tag{S2.1}$$

Figure S2.1a shows the relation between the flight direction θ and the ground speed (the magnitude of ground velocity) $|\vec{v}|$ for one flight data in dataset D1. The ground speed varies with θ sinusoidally. This sinusoidal modulation should result from the wind effect.

By adopting a few assumptions, we can estimate the wind velocity and the airspeed of birds. Here, we assume the following two conditions: (a1) $\vec{w}$ = const., i.e., the wind is steady and uniform during each flight data; (a2) $|\vec{v}^a|$ = const., i.e., airspeed of each bird is constant.

Actually, by taking the absolute value of both sides in Eq. (S2.1), we obtain following equation for ground speed:

$$|\vec{v}| = |\vec{v}^a| \sqrt{1 + 2\frac{|\vec{w}|}{|\vec{v}^a|}\cos\Delta + \frac{|\vec{w}|^2}{|\vec{v}^a|^2}}, \tag{S2.2}$$

where Δ denotes the angle between $\vec{v}^a$ and $\vec{w}$. If we take into account third assumption (a3) the wind speed is much slower than airspeed, i.e. $|\vec{w}| \ll |\vec{v}^a|$, we obtain

$$|\vec{v}| \approx |\vec{v}^a| + |\vec{w}|\cos\Delta', \tag{S2.3}$$

with Δ' is the angle between $\vec{v}$ and $\vec{w}$. Assumptions (a1), (a2) and (a3) induce the sinusoidal modulation of $|\vec{v}|$ with respect to θ shown in Figure S2.1a.

However, by assuming (a1) and (a2) only, one can estimate the wind velocity and airspeed without (a3), by searching three constant values $(|\vec{v}^a|, \vec{w})$ which minimise the quantity:

$$D = (|\vec{v} - \vec{w}|^2 - |\vec{v}^a|^2)^2. \tag{S2.4}$$

In order to perform the estimation procedure, we treat each flight dataset separately to satisfy the condition (a1). By using the Levenberg-Marquardt method for each individual's flight time series, we estimate the wind velocity during the flight and the airspeed of each pigeon independently. Figure S2.1b exhibits typical results of the wind velocity estimation. Different colours correspond to different flight data observed in different day. The arrows with same colours indicate the estimated wind velocity by different pigeon's in the same flight. The statistical value of the same colour arrows is summarised in Table S2.1. The estimated wind velocities well resemble each other in the same flight data (colour).

Note that in the main text, we use only the averaged ground velocity for 20 sec, a typical period for one cycle in circling motion. We confirm that obtained results have no significant difference between using the averaged ground speed and the airspeed for circling flights (Figure S2.1c).

Finally, we comment on the assumption (a2): In order to fly along a desired orbit, pigeons may control both their speed $|\vec{v}^a|$ or their moving direction. Therefore the condition (a2) is an extreme assumption. And the validity of this assumption remains open. Further studies are required to clarify whether this assumption is valid or not.

Reference

[1] H.U.Roll, Physics of the marine atmosphere, 1965 Academic Press, p.226.

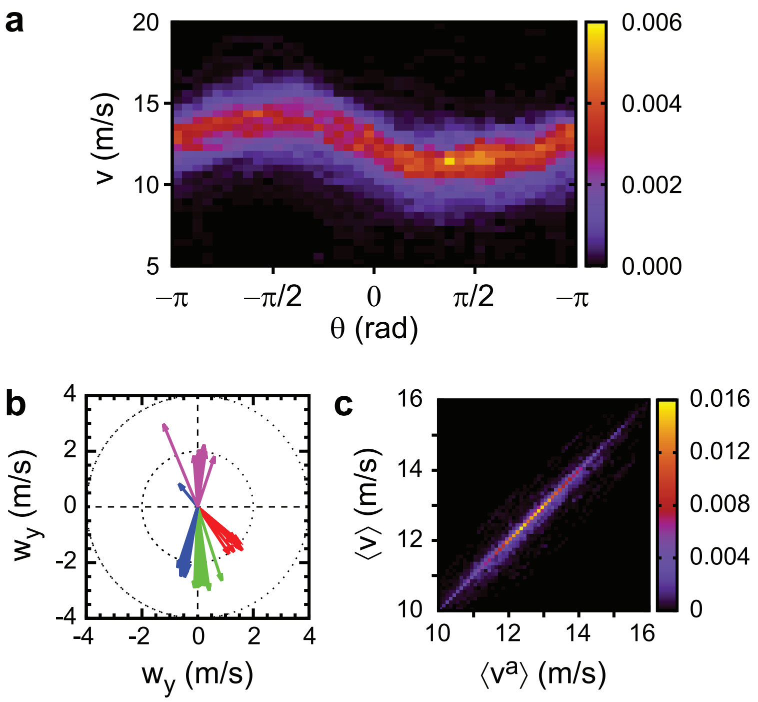

Figure S2.1. Estimation of the wind. (a) Typical example of the relation between the ground speed of the pigeons and the flight direction. Sinusoidal modulation is observed. (b) Typical results of the wind velocity estimation. Different colours correspond to the different flight, and the arrows with the same coluor indicate the estimated wind velocity by different individuals in the same flights. (c) The relation between the averaged airspeed $\langle v^a \rangle$ and the averaged ground speed $\langle v \rangle$. The former is calculated by subtracting the estimated wind velocity from the ground velocity.

Table S2.1. Statistical value of the estimated wind velocity.

flight dataset	Data	Number	w_x (m/s)	w_y (m/s)	$\lvert\vec{w}\rvert$ (m/s)	c_v	colour
D1-1	26th August	8	1.39 ± 0.15	-1.42 ± 0.19	1.99 ± 0.16	0.996	red
D1-2	24th September	10	0.01 ± 0.48	2.03 ± 0.37	2.0 ± 0.43	0.981	magenta
D1-3	25th September	9	0.12 ± 0.39	-2.48 ± 1.00	2.54 ± 0.90	0.976	green
D1-4	26th September	10	-0.56 ± 0.12	-1.95 ± 0.99	2.20 ± 0.43	0.921	blue

The number indicates the number of individuals, i.e., the number of the arrows in Figure S2.1b. w_x and w_y are the estimated wind velocity components of each direction with their standard deviations. $|\vec{w}|$ (± s.d.) is the *scalar average* of the wind velocity, i.e., average of the magnitude of the wind velocity. The parameter c_v is an analogue of the quantity of "constancy" of the general aperiodic wind variation [1]. It is defined as the ratio of the magnitude of the average vector wind velocity to the average scalar wind velocity, i.e., $c_v = \sqrt{\langle w_x \rangle^2 + \langle w_y \rangle^2} / \langle |\vec{w}| \rangle$, where the $\langle \, \rangle$ denotes the time average. If the wind vector is constant in time, c_v is equal to the unity. On the other hand c_v vanishes if wind direction is isotropic. We apply this quantity to represent how the estimated wind vector by each bird resembles each other by changing its averaging operation not in time but for individuals. Actually, all c_v values is almost unity. The colour corresponds to the colour of the arrows in Figure S2.1b.
